# Cultural Competence Interventions for Health Care Providers Working With Racialized Foreign-born Older Adults: Protocol for a Systematic Review

**DOI:** 10.2196/31691

**Published:** 2022-07-26

**Authors:** Diya Chowdhury, Leonardo Baiocco-Romano, Veronica Sacco, Karen El Hajj, Paul Stolee

**Affiliations:** 1 University of Waterloo Waterloo, ON Canada

**Keywords:** cultural competence, racialized older adults, person-centered care, immigrants, health systems interventions

## Abstract

**Background:**

Integrating culturally competent approaches in the provision of health care services is recognized as a promising strategy for improving health outcomes for racially and ethnically diverse populations. Person-centered care, which ensures patient values guide care delivery, necessitates cultural competence of health care providers to reduce racial/ethnic health disparities. Previous work has focused on interventions to improve cultural competence among health care workers generally; however, little investigation has been undertaken regarding current practices focused on racialized foreign-born older adults.

**Objective:**

We seek to synthesize evidence from existing literature in the field to gain a comprehensive understanding of interventions to improve the cultural competence of health professionals who care for racialized foreign-born older adults. The aim of this paper is to outline a protocol for a systematic review of available published evidence.

**Methods:**

Our protocol will follow the PRISMA-P (Preferred Reporting Items for Systematic Reviews and Meta-Analyses–Protocols) for systematic review protocols. We will conduct a systematic search for relevant studies from four electronic databases that focus on health and social sciences (PubMed, CINAHL, Scopus, and Cochrane Database). After selecting relevant papers using the inclusion and exclusion criteria, data will be extracted, analyzed, and synthesized to yield recommendations for practice and for future research.

**Results:**

The systematic review is currently at the search phase where authors are refining the search strings for the selected databases; the search strings will be finalized by July 2022. We anticipate the systematic review to be completed by December 2022.

**Conclusions:**

This study will inform the future development and implementation of interventions to support culturally competent, person-centered care of racialized foreign-born older adults.

**Trial Registration:**

PROSPERO CRD42021259979; https://www.crd.york.ac.uk/prospero/display_record.php?RecordID=259979

**International Registered Report Identifier (IRRID):**

PRR1-10.2196/31691

## Introduction

### Background

Recent changes in population demographics, as a result of increased migration across national borders, have led to the reconsideration of traditional health care practices [1-3]. The rapid increase in migration and globalization patterns in high-income countries has important implications for health systems, health care workers, and the health of individuals [4,5]. Health and quality of life consequences for immigrants of racial and ethnic disparities in health care have been well documented [2,6,7]. Challenges faced by racialized foreign-born older adults (FBOAs) include greater difficulty accessing health services, lower likelihood of routine services, and an overall lower quality of care [8-13]. For FBOAs, access and quality of services is of particular concern [3,14]. Studies assessing the disparities in health care and health outcomes among racialized groups, including older adults, have identified race, ethnicity, and cultural variables as predictors of poorer health outcomes [4,14,15]. Health status disparities result in higher rates of mortality and morbidity among FBOAs even though immigrants report better health outcomes compared to non-FBOAs upon arrival [4,16]. The lack of clarity in health care systems on how to best cater social and health services for FBOAs further exacerbates health disparities [4,13,17].

Increased cultural competency of health care professionals is recognized as important for improving the provision of health care for racial/ethnic minority groups [7,18,19]. For older adults, it has been recognized that cultural competence is essential to meet the needs of what is becoming a larger and more diverse population [20]. The value of cultural competence lies in understanding how cultural variables impact and inform the health care experiences of older adults [21,22]. However, early literature commonly framed cultural competence as a list of “dos and don’ts” that may result in stereotypical thinking [14,23,24]. Instead, health care workers should view cultural competence as a core component of clinical competence [21,24].

There is increasing recognition of the importance of including patient perspectives on the quality of health care delivery, which has traditionally been driven solely by health care professionals and policy makers [23,25]. Consequently, person-centered care, which prioritizes viewing older adults as partners in receiving, planning, and monitoring care, has received significant traction over the last few decades [26,27]. Ensuring patients are involved and central to their care is now recognized as a key component of supporting high-quality health care [26,27]. Because older adults from culturally diverse backgrounds are often miscategorized as a homogenous group despite the prominent sociocultural differences that exist among them, person-centered care merits considerable attention in the care of FBOAs [28]. By moving away from a “one-size-fits-all” approach, person-centered care necessitates that care providers tailor care delivery to the participants’ specific sociocultural backgrounds [26,27]. As such, person-centered care is a crucial component of cultural competence in health care settings, and each informs the other [29,30].

Several systematic reviews have been conducted on cultural competency in various care settings and within various disease modalities. One such review sought to compile the perceptions of culturally competent care for lesbian, gay, bisexual, and transgender (LGBT) long-term care (LTC) residents [31]. It was found that staff lack training when caring for LGBT residents in LTC and report negative feelings toward same-sex relationships among older adults [31]. Similarly, in a study assessing the impact of being a language minority in LTC, Batista and colleagues [32] reported that the capacity to deliver care in residents’ languages could impact health outcomes. Reviews on other populations beyond racialized FBOAs found that culturally competent care, including the use of interpreters, staff cultural training, and culturally appropriate training, could reduce racial and ethnic disparities [31].

Truong and colleagues [33] conducted a systematic review of reviews that sought to synthesize existing reviews investigating the effectiveness of cultural competence interventions. They found, within a broad array of contexts and study types, that interventions addressing cultural competence produced moderate improvements in provider and health care access outcomes, and weak improvements in patient outcomes [33]. This review, however, did not distinguish between foreign-born and non–foreign-born older adults [33]. This highlights the need for a systematic review that emphasizes the role of health care providers in managing and improving the health of racialized FBOAs and the need for further investigation surrounding culturally competent care in the health care system [34].

We propose to look at all interventions pertaining to culturally competent practices and training for health care professionals working with FBOAs. This will allow us to better understand the merit of existing interventions and highlight similarities and differences with relevant implications for care provided. Both culture and competence are multidimensional and multifactorial concepts [35,36]. As a result, several different terminologies such as cultural competence, cultural safety, or multi- or cross-cultural competence, are used interchangeably owing to the lack of consistent and clear definitions [33,37-39]. Consequently, health care professionals view the term through the lens of their respective disciplines [33,37,38]. For the purposes of our review, the widely used definition of cultural competence provided by Cross and colleagues [40] will be used, that being, “a set of congruent behaviors, attitudes, and policies that come together in a system, agency, or among professionals and enables that system, agency, or those professionals to work effectively in cross-cultural situations.” Additionally, the definition of cultural competence interventions provided by Truong and colleagues [33] will be used, that being, any intervention that aims to improve health care effectiveness and accessibility for people belonging to racial or ethnic minorities by increasing the provider and patient knowledge, skills, or awareness. As a byproduct of this synthesis, we aim to reach consensus and refine the definition of cultural competence and gain a better understanding of what cultural competence entails specifically relating to FBOAs. The overall aim of the proposed systematic review is to synthesize current best practices regarding interventions to promote cultural competence for health care professionals working with FBOAs.

### Study Rationale

Cultural competence allows health care professionals to account for the specific cultural contexts of older adults from different ethnic and racial backgrounds [14,18,19]. In recent years, substantial evidence from public health reports and research findings indicated that racialized immigrants are underserved and have a higher likelihood of receiving negative and differential health outcomes compared to their nonimmigrant counterparts [4,13,17]. In response, health systems in countries with high immigrant populations have attempted to incorporate cultural competence in their health delivery practices and policies to improve quality of care for racially and ethnically diverse populations [8,41,42]. Although racialized FBOAs account for a large portion of the overall population in these countries, published research for understanding the needs of this subpopulation, particularly older adults, is limited [8]. We anticipate most of these studies will come from western and high-income countries, but we do not wish to limit or exclude studies from low-income countries. Despite culturally competent interventions showing promise in promoting positive health outcomes, there has been a lack of recent systematic appraisal of its impact for racialized FBOAs [43]. As such, the review will not be limited to the interventions conducted in any one country or by the origins of the FBOAs under study within the studies. Any older adult belonging to a racialized group who have migrated to a country outside of their birth country will be considered. Any intervention aiming to improve the quality of cultural competence within the practitioners caring for those FBOAs will be considered.

### Study Objective

To improve health outcomes of older adults, it is important to understand if health care systems reinforce health disparities, including assessment of how inadequate services affect the well-being of racialized FBOAs. To do so, we need to understand the impact of culture on health care experiences, delivery, and planning; whether the unique health care needs of ethnic and racial older adults are being adequately met; and the merits of provider cultural competence interventions for racialized FBOAs. This paper will outline the protocol for a systematic review that will review all interventions pertaining to culturally competent practices and training for health care professionals working with racialized FBOAs that have been implemented across various care settings. This will allow us to better understand the value of existing interventions and highlight similarities and differences with relevant implications for care provided.

## Methods

### Protocol Design

Our systematic review protocol (PROSPERO registration CRD42021259979) follows the PRISMA-P (Preferred Reporting Items for Systematic Reviews and Meta-Analyses–Protocols) reporting guidelines [44].

### Eligibility Criteria

The systematic review will consider all relevant health interventions aimed to improve culturally competent care for FBOAs throughout the health system. The papers selected will include peer-reviewed publications published in English until December 31, 2021. Prior reviews regarding cultural competence interventions only included articles published after the year 2000, when cultural competence started to gain recognition as an issue of concern in the health care field [33]. To capture a broader range of information, as well as information that may have served as a prelude to the recognition of cultural competence as a necessity in health care, no early date limit will be used. Papers selected for the review will include qualitative, quantitative, and mixed methods studies that describe and apply culturally competent practices across health care settings. The review will consider a variety of health settings that include but are not limited to LTC, hospital care, and home and community care. The inclusion of a wide range of health settings helps inform the trajectory and progress of culturally competent care throughout the health literature. Following the World Health Organization’s guidelines, we will use the age range of >60 years to define older adults [45]. Following Ontario’s Human Rights Code, we use the term “racialized” to describe persons of color and visible minority populations [46-48]. “Foreign-born” persons include those who were born in a country that is different than the one they reside in, as defined by the Organisation for Economic Co-operation and Development, the US Census Bureau, and Ontario’s Human Rights Code [46-48]. Our selected population will include older adults (aged ≥60 years) who are racialized FBOAs. To be included, a study must consider the care of a racialized FBOA by any health care provider. For the purposes of this paper, health care providers include health care professionals or other paid health care workers who work in any community or institutional health care setting, including hospitals and LTC homes. Studies focusing on adults who are not 60 years or older will be excluded, as well as studies that do not consider cultural competence interventions given to health care providers. All studies not written in English and all gray literature will not be included.

### Search Methods

A systematic search of the published literature will be performed using defined search terms. The systematic review will use four databases (PubMed, CINAHL, Scopus, and the Cochrane Database) to search for peer-reviewed articles, with dates ranging from inception to December 2021. Selected databases and search strategies have been developed by all authors with the guidance of a health sciences librarian.

The search strategy for the review was developed for PubMed and will be used as a template for the remaining databases. The search strategy will use Medical Subject Headings (MeSH) terms and keywords identified in relevant papers. In adherence to the PRISMA-P guidelines, the protocol paper describes a draft of the search string as well as the number of results obtained [44]. The initial search results that have been developed for PubMed, as of April 25, 2022, are shown in Textbox 1. The titles and abstracts of articles will be used for the initial screening. Upon completing screening for abstracts and full papers, reference lists of selected journal articles will be hand searched for additional papers.

**PubMed search strategy (762 PubMed search results).**

**Cultural competence**

(“cultural competenc*”[Text Word] OR “cultural diversity*”[Text Word] OR “cultural appropriateness*”[Text Word] OR “cultural responsiveness*”[Text Word] OR “cultural sensitivity*”[Text Word] OR “multicultural education”[Text Word] OR “cultural self-efficacy”[Text Word])

**Older Adults**

(“elder*”[Text Word] OR “senior*”[Text Word] OR “older adults”[Text Word] OR “aged”[MeSH Terms]) 

**Racialized foreign-born**

(“emigration and immigration”[MeSH Terms] OR “ethnicity”[MeSH Terms] OR “ethnic group”[Text Word] OR “refugee*”[Text Word] OR “newcomer*”[Text Word] OR “migrant*”[Text Word] OR “immigrant*”[Text Word])

The final search strategy prepared for this systematic review will be completed and recorded on June 28, 2022. Study selection on Covidence [49] will begin on July 4, 2022, to allow for some time to review the retrieved articles, prepare the study selection tool, and train reviewers to apply the inclusion and exclusion criteria. It is expected that title and abstract selection will take 1 month, at which point full-text selection will begin and last for approximately 3 weeks. Following study selection, data extraction and risk of bias assessments will be conducted and are expected to take an additional month to complete. It is expected that data synthesis and reporting will begin in August 2022 and will take approximately 2 months to complete, at which point discussion and finalization of the report will take an additional month to complete. Following this, the final systematic review will be submitted for publication in a journal relating to public health by the end of 2022.

### Data Collection and Analysis

#### Selection of Studies

This review will consist of a two-stage article screening procedure. Results obtained from our search will be imported into Covidence, a web-based software that facilitates systematic review management [49]. Two pairs of reviewers will independently screen each identified article’s titles and abstracts based on the predetermined inclusion and exclusion criteria. Reviewers will be blinded to each other’s decisions. Discrepancies will be resolved through discussion among authors to reach a final decision, and articles deemed irrelevant by the majority (at least three of the four reviewers) will be eliminated from the review. Reasons for excluding articles will be noted throughout the process.

Following title and abstract review, two pairs or two reviewers will independently conduct full-text screening to finalize the included articles prior to data extraction. Any emerging disagreements between reviewers will be resolved by consulting the other coauthors. Furthermore, a group training session will be held prior to screening to ensure that all authors follow a consistent approach when screening studies. This group session will include an overview of the mechanics of Covidence, and a discussion of how to consistently apply the inclusion and exclusion criteria. The results of the previously mentioned steps will be recorded and presented according to the PRISMA-P flow diagram [50].

#### Data Extraction and Management

Data extraction will proceed separately for quantitative and qualitative studies (see Multimedia Appendices 1 and 2 for sample extraction tables [51,52]). If mixed methods studies are identified, a separate extraction table will be created based on the main categories described below. The review team, consisting of four of the reviewing authors, will be trained on extraction categories using Cochrane’s training resources [53]. All reviewers will pilot test the data extraction table to ensure consistency. This process will ensure that reviewers are extracting similar types of data. Any discrepancies that arise throughout the extraction process will be discussed by the team and documented. If necessary, the extraction form will be adjusted to reflect team decisions, and a second sample of research studies will be reviewed to ensure reviewers are extracting similar data. Any discrepancies that arise throughout the extraction process will be discussed by the team and documented. If necessary, the extraction form will be adjusted to reflect team decisions, and a second sample of research studies will be reviewed to ensure reviewers are extracting similar data. From selected studies, we expect the following overarching information will be extracted: study identification items (ie, authors and year of publication), study characteristics (eg, country, care setting, and participant characteristics), study design and methods, findings/results, and definition of cultural competence. The extracted data from the papers will be compiled into data sheets independently by all four authors. The reviewers will then compare data sheets and as a group compile a final extraction table. Any discrepancies will be resolved by consulting other coauthors.

#### Outcomes 

Due to the likely heterogeneous nature of the studies to be included within the review, target outcome measures were not specified a priori. However, following Bronfenbrenner’s Ecological-Environmental model, we expect that, for both qualitative and quantitative studies, outcomes can be broadly classified under patient (micro-level), organization (meso-level), or system-level outcomes (macro-level) [54]. The studies are unlikely to be statistically comparable, since we expect the interventions and outcomes to be disparate, but we will consider outcomes and impacts at each of the specified levels.

#### Assessing Bias

To assess the risk of bias in quantitative studies included within the review, the Risk of Bias 2 (ROB2) and Risk of Bias In Non-randomized Studies of Interventions (ROBINS-1) tools will be used [55,56]. The ROB2 tool was designed to assess the risk of bias in randomized trials along five domains: bias arising from the randomization process, bias due to deviations from intended interventions, bias due to missing outcome data, bias in measurement of the outcome, and bias in the selection of the reported result [55]. The ROBINS-1 tool is used to assess risk of bias in nonrandomized studies along seven domains: bias due to confounding, bias in selection of participants, bias in classification of interventions, bias due to deviations from intended interventions, bias due to missing data, bias in measurement of outcomes, and bias in the selection of the reported result [56].

#### Quality Assessment

Quantitative studies included within the review will be assessed using The Effective Public Health Practice Project Quality Assessment Tool for Quantitative Studies, which is a validated tool designed to assess the quality of articles within systematic reviews [57]. This tool assesses eight indicators—selection bias, study design, confounders, blinding, data collection methods, withdrawals and dropouts, intervention integrity, and analysis—and rates items as either strong, moderate, or weak [57]. These ratings are then aggregated to create an overall rating of each paper to be listed under one of three categories [57]. This tool was selected because it allows for a broad array of studies to be assessed. Two reviewers will independently assess each article that meets inclusion criteria, and in the case of a disagreement, a third author will assess the study.

Qualitative studies selected for the review will be assessed using the Critical Appraisal Skills Programme Qualitative Checklist [58]. This appraisal tool consists of 10 questions and can be used to determine the strengths and limitations of different qualitative studies [58]. Two reviewers will independently assess selected articles, and in the case of a disagreement, a third author will provide input.

Mixed methods studies selected for review will be assessed for methodological validity by two independent reviewers according to the procedures outlined by the Methodology for Joanna Briggs Institute Mixed Methods Systematic Reviews [59]. Any disagreements that arise between reviewers will be resolved by consulting a third author.

### Ethical Considerations

Since the systematic review will involve data collection from publicly available resources, this study will not require ethics approval.

## Results

The results will be reported according to the outcomes specified above. We anticipate that our findings will be useful for those aiming to develop or implement interventions that support culturally competent care for racialized FBOAs and as a basis for future research. Results of the review will be disseminated widely, and the review will be submitted for publication at a peer-reviewed journal.

## Discussion

We expect that this systematic review will comprehensively synthesize the existing literature surrounding interventions aimed at improving the cultural competence of health care professionals who care for racialized FBOAs. We anticipate that the findings will be heterogeneous but will nonetheless prove valuable in highlighting interventions that improve the competence of health care professionals, from both a quantitative and qualitative perspective. The results of this systematic review will aid in program planning and training of health care professionals who care for racialized FBOAs by providing evidence for effective strategies to increase cultural competence. The final manuscript produced for this study will be disseminated through a peer-reviewed academic journal and will be distributed widely to stakeholders within the health care field.

## Appendix

Multimedia Appendix 1 Quantitative data extraction sheet.

Multimedia Appendix 2 Qualitative data extraction sheet.

## Abbreviations

FBOA: foreign-born older adult
LGBT: lesbian, gay, bisexual, and transgender
LTC: long-term care
MeSH: Medical Subject Headings
PRISMA-P: Preferred Reporting Items for Systematic Reviews and Meta-Analyses–Protocols
ROB2: Risk of Bias 2
ROBINS-1: Risk of Bias In Non-randomized Studies of Interventions
