# Supplementary material for: Cultural Competence Interventions for Health Care Providers Working With Racialized Foreign-born Older Adults: Protocol for a Systematic Review
Source: JMIR Res Protoc. 2022 Jul 26;11(7):e31691. doi: 10.2196/31691 (PMC9364170; doi:10.2196/31691)
Supplement: Multimedia Appendix 1 [file resprot_v11i7e31691_app1.docx]

**Appendix 1:**

**Quantitative Data Extraction Sheet**

Reviewer name:

Date:

| **Study Identification** | | | | **Context and Participants** | | |
| --- | --- | --- | --- | --- | --- | --- |
| *Article number* | *DOI* | *Authors* | *Year of publication* | *Country* | *Study setting* | *Participant characteristics* |
|  |  |  |  |  |  |  |
|  |  |  |  |  |  |  |
|  |  |  |  |  |  |  |

| **Study Design and Methods** | | | | | | |
| --- | --- | --- | --- | --- | --- | --- |
| *Article number* | *Study aims* | *Research questions* | *Study design* | *Recruitment procedure* | *Intervention details* | *Definition of cultural competence* |
|  |  |  |  |  |  |  |
|  |  |  |  |  |  |  |
|  |  |  |  |  |  |  |

| **Study Result** | | | |
| --- | --- | --- | --- |
| *Article number* | *Outcome scale (if applicable)* | *Outcome measure* | *Intervention results* |
|  |  |  |  |
|  |  |  |  |
|  |  |  |  |
