# Supplementary material for: Cultural Competence Interventions for Health Care Providers Working With Racialized Foreign-born Older Adults: Protocol for a Systematic Review
Source: JMIR Res Protoc. 2022 Jul 26;11(7):e31691. doi: 10.2196/31691 (PMC9364170; doi:10.2196/31691)
Supplement: Multimedia Appendix 2 [file resprot_v11i7e31691_app2.docx]

**Appendix 2:**

**Qualitative Data Extraction Sheet**

Reviewer name:

Date:

| **Study Identification** | | | | **Context and Participants** | | |
| --- | --- | --- | --- | --- | --- | --- |
| *Article number* | *DOI* | *Authors* | *Year of publication* | *Country* | *Care setting* | *Participant characteristics* |
|  |  |  |  |  |  |  |
|  |  |  |  |  |  |  |
|  |  |  |  |  |  |  |
|  |  |  |  |  |  |  |

|  | **Study Design and Methods** | | | | | | |
| --- | --- | --- | --- | --- | --- | --- | --- |
| *Article number* | *Study aims* | *Research questions* | *Study type (methodological approach)* | *Recruitment procedure* | *Data collection methods* | *Data analysis approach* | *Definition of cultural competence* |
|  |  |  |  |  |  |  |  |
|  |  |  |  |  |  |  |  |
|  |  |  |  |  |  |  |  |

|  | **Study Findings** | | **Quality of the study** |
| --- | --- | --- | --- |
| *Article number* | *Key themes identified* | *Recommendations made by authors* |  |
|  |  |  |  |
|  |  |  |  |
|  |  |  |  |

The data extraction sheet was informed by Noyes & Lewin [58] and Munro et al. [59].
